# Disproportionality Analysis of Hematologic Adverse Event Signals Associated with Venetoclax in Combination with Senescence-Inducing Chemotherapy

**DOI:** 10.3390/jcm15062194

**Published:** 2026-03-13

**Authors:** Tareq Saleh, Mohannad Ramadan, Anoud Alsoud, Sofian Al Shboul

**Affiliations:** 1 Department of Pharmacology & Therapeutics, College of Medicine & Health Sciences, Arabian Gulf University, Manama 26671, Bahrain; 2 Department of Pharmacology and Public Health, Faculty of Medicine, The Hashemite University, Zarqa 13133, Jordan

**Keywords:** venetoclax, navitoclax, senolytics, therapy-induced senescence, FAERS

## Abstract

**Background**: BH3 mimetics (such as venetoclax and navitoclax) are increasingly investigated in the context of the “one-two punch” anticancer strategy, wherein senescence-inducing therapies are combined with senolytic clearance. However, real-world pharmacovigilance evidence describing hematologic adverse event (AE) patterns and serious outcomes for venetoclax versus navitoclax in such combination settings remains limited. This study aims at providing an expectation based on the current reporting of the safety implications of senolytics combined with senescence-inducing therapy in clinical practice. **Methods**: We analyzed de-duplicated U.S. FDA Adverse Event Reporting System (FAERS) reports retrieved on 1 August 2025. Venetoclax reports (Q2 2016–Q2 2025) were categorized as monotherapy or combination with senescence-inducing chemotherapy (predefined based on published evidence of therapy-induced senescence [TIS]). Hematologic AEs were grouped into three categories (isolated low WBC, isolated low platelet count, and multi-lineage cytopenia). Disproportionality analyses were conducted using the Reporting Odds Ratio (ROR) and Proportional Reporting Ratio (PRR) with 95% CIs and chi-squared testing. Navitoclax reports were analyzed descriptively due to limited volume. **Results**: A total of 47,508 venetoclax reports were included (34,485 monotherapy; 13,023 combination). Compared with monotherapy, combination therapy showed disproportionate reporting signals (ROR/PRR; reflecting reporting disproportionality rather than incidence or causal risk) for low WBC (ROR 2.87, PRR 2.59) and multi-lineage cytopenias (ROR 3.54, PRR 2.94), while isolated low platelet count was under-represented (ROR 0.31, PRR 0.32). For outcomes, combination therapy demonstrated higher reporting signals for life-threatening outcomes (ROR 7.06, PRR 6.56), hospitalization (ROR 1.74, PRR 1.39), and other outcomes (ROR 2.36, PRR 1.57), while death (ROR 0.55, PRR 0.65) and non-serious outcomes (ROR 0.26, PRR 0.29) were proportionally less reported (all *p* < 0.001). Navitoclax had 172 reports; hematologic cytopenias and serious outcomes were frequent, but analyses were descriptive only. **Conclusions**: In FAERS, venetoclax combined with senescence-inducing chemotherapy shows stronger reporting signals for leukopenia and multi-lineage cytopenias and for several serious outcome categories compared with monotherapy. These reporting patterns highlight the need for further care in terms of clinical implementation of the currently investigated senolytics prior to the consideration of the “one-two punch” strategy.

## 1. Introduction

Chemotherapy can exert its effect on tumor cells, in part, by inflicting therapy-induced senescence (TIS) [1]. TIS can be precipitated by a plethora of conventional or targeted therapies, forcing tumor cells into a state of senescence-associated growth arrest (SAGA), which has been traditionally viewed as a permanent state [2]. The irreversibility of the SAGA accounts largely for its tumor-suppressive role of TIS [3]. However, newer evidence has demonstrated that SAGA is not obligatorily permanent, and tumor cells can escape and re-enter the cell cycle to resume proliferation, events that could eventually contribute to therapy resistance and cancer recurrence [1]. Moreover, TIS has been implicated in mediating several therapy-associated adverse effects, primarily through its secretory phenotype (the SASP) that could impact the course of cancer treatment [4]. These untoward contributions of TIS in cancer treatment provided the basis for considering the strategy of eliminating senescent tumor cells using selective pharmacological agents (i.e., senolytics) [4].

Senolytics are a group of agents that target senescent cells through several vulnerabilities, including a primary category that inhibits Bcl-2 family anti-apoptotic proteins (Bcl-2, Bcl-xL, Bcl-w, and Mcl-1) [5]. Navitoclax (ABT-263) is the first orally bioavailable BH3 mimetic with a high affinity for Bcl-2, Bcl-xL, and Bcl-w (non-selective), which interferes with their anti-apoptotic role [6]. Navitoclax can both compete with and displace the BH3 pro-apoptotic proteins, and as a result, drives cells into apoptosis [6]. A number of hematolymphoid malignancies, such as chronic lymphocytic leukemia (CLL), small lymphocytic lymphoma (SLL), follicular lymphoma (FL), diffuse large B-cell lymphoma (DLBCL), and acute lymphoblastic leukemia (ALL), have shown experimental response to navitoclax in various degrees [7]. Also, in preliminary studies, navitoclax has demonstrated effectiveness against specific solid tumors such as prostate, lung, breast, and colon cancers, primarily due to Bcl-xL inhibition, especially when combined with other chemotherapies [8]. However, due to its high affinity for Bcl-xL, which is essential for platelets’ survival, navitoclax was reported to cause severe dose-limiting thrombocytopenia, which constrained its use, especially as monotherapy [9]. However, navitoclax has recently emerged as a very potent senolytic in many aging-related and tumor cell models, which invited for its repurposing [10,11,12].

Venetoclax is an orally bioavailable BH3 mimetic distinguished by its high selectivity and affinity for Bcl-2 while sparing Bcl-xL and other anti-apoptotic proteins [13]. This selectivity led to two important findings: first, venetoclax resolved, in part, the platelet toxicity issue that was encountered with navitoclax; second, venetoclax is mostly used with hematolymphoid malignancies, as the Bcl-2 protein is especially overexpressed in these tumors, unlike solid tumors, which more commonly express Bcl-xL and Mcl-1 [14]. Venetoclax is FDA-approved as a monotherapy in patients with CLL with del(17p) after at least one prior therapy or in combination with other types of chemotherapy like rituximab or obinutuzumab, both of which can result in TIS [7,15]. Furthermore, it is approved in acute myeloid leukemia (AML) in adults older than 75 years or unfit to intensive induction due to comorbidities [7,15]. In this case, venetoclax can be combined with azacitidine, decitabine, or low-dose cytarabine (LDAC), all of which are senescence-inducing therapies [7]. Experimentally, it has exhibited response in a range of lymphoid malignancies (e.g., mantle cell lymphoma and Waldenström macroglobulinemia) [7]. Importantly, these venetoclax-based regimens in CLL and AML were developed and established primarily as standard-of-care treatment strategies rather than being specifically implemented as a senolytic combinatory treatment.

Although venetoclax overcame dose-limiting platelet toxicity, it is less potent as a senolytic when compared to navitoclax [12]. In preclinical studies, navitoclax has been reported to show greater senolytic activity than venetoclax in some hematological models, especially ALL [16], and in solid tumors such as glioblastoma [17], triple-negative breast cancer (TNBC), and lung adenocarcinoma [18]. This is largely because senescent tumor cell survival is dependent on Bcl-xL, which is not targeted by venetoclax [14,19]. Nevertheless, some of the emerging evidence suggests that venetoclax might be equally effective as navitoclax as a senolytic in certain tumor cell models, especially when combined with senescence-inducing therapy in vivo [20,21,22,23]. In all cases, the use of venetoclax would be more favorable since it is associated with fewer hematological adverse effects (AEs). Still, an investigation of the AE frequency of both agents in combination with senescence-inducing chemotherapy, which potentially will be the ideal approach for their use as the “one-two punch” approach [24], has not been directly evaluated using FAERS-based monotherapy versus combination disproportionality comparisons. In particular, it is unclear whether combining senolytics with senescence-inducing chemotherapy regimens modifies the hematologic AE profile or serious outcome patterns compared with senolytic monotherapy. To address this gap, we evaluated pharmacovigilance reports to determine whether AE reporting patterns differ between monotherapy and combination therapy, thereby testing the hypothesis that implementing senolytic–senescence-inducing chemotherapy pairing in clinical practice would be associated with a distinct safety signal profile.

## 2. Materials and Methods

### 2.1. Data Source

**Figure 1:**
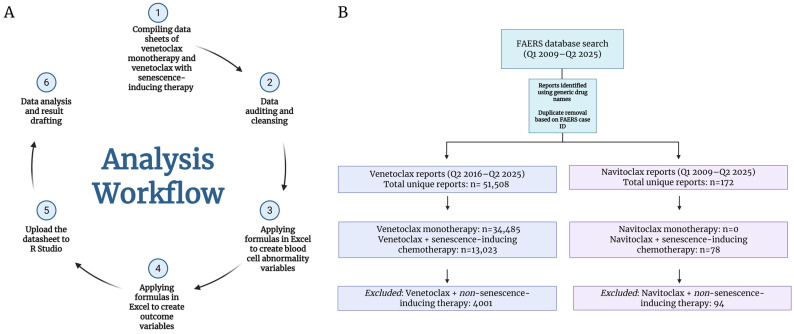
Workflow of the analysis. (**A**) This flow chart illustrates the sequential steps undertaken for data preparation, variable derivation, and statistical analysis. Initially, raw datasheets were compiled for patients receiving venetoclax monotherapy and venetoclax in combination with senescence-inducing therapy. The compiled datasets then underwent systematic data auditing and cleansing to identify and correct inconsistencies, missing values, and entry errors. Following data cleaning, predefined formulas were applied in Microsoft Excel to derive blood cell abnormality variables based on MedDRA preferred-term membership rules. Subsequently, additional Excel-based formulas were used to generate outcome variables according to the study’s clinical and analytical definitions. The finalized dataset was then uploaded into R Studio for statistical processing. The workflow concludes with data analysis and result drafting, including the generation of summary statistics, comparative analyses, and figures for reporting. Created in BioRender. (2026) https://BioRender.com/qn6hiv2. (**B**) FAERS reports were identified using generic drug names and de-duplicated by case ID. A total of 51,508 unique venetoclax reports (Q2 2016–Q2 2025) and 172 navitoclax reports (Q1 2009–Q2 2025) were retrieved. Venetoclax reports included monotherapy and combinations with senescence-inducing chemotherapies, while reports involving non-senescence-inducing combinations were excluded. Created in BioRender. (2026) https://BioRender.com/4xsdzif.

For this pharmacovigilance study, we used the U.S. FDA Adverse Event Reporting System (FAERS) [25]. All available FAERS reports that mentioned venetoclax or navitoclax were retrieved on 1 August 2025. Reports released by the FAERS website after the retrieval date were not included in our analysis. Venetoclax was FDA-approved in April 2016 [26], whereas navitoclax did not get FDA approval due to its associated platelet toxicity. Reports were identified using generic names of agents, and duplicate cases were removed based on the FAERS case identifier (case ID), retaining unique reports for analysis (Figure 1A). Reports were considered eligible if venetoclax or navitoclax was listed in the FAERS drug records, regardless of case role classification. Accordingly, the main analysis included reports in which the study drug appeared under any available role designation (e.g., primary suspect, secondary suspect, concomitant, or interacting, where present in the FAERS export), and no restriction to primary suspect reports was applied. This operational definition was used for both venetoclax and navitoclax datasets prior to de-duplication and downstream categorization. After de-duplication, 51,508 venetoclax reports were initially retrieved from the FAERS between Q2 2016 and Q2 2025. Following application of the study-specific inclusion and categorization workflow (including classification into monotherapy (34,485 reports) versus combination with predefined senescence-inducing therapy (13,023 reports) and exclusion of reports not retained in the final analytic dataset (4000 reports)), 47,508 venetoclax reports were included in the final analysis (Figure 1B). For navitoclax, 172 unique reports were retrieved between Q1 2009 and Q2 2025; none reported navitoclax as monotherapy, while 78 reported using it in combination with one of the senescence-inducing chemotherapies (Figure 1B). 

### 2.2. Determination of Senescence-Inducing Chemotherapy Used in Combination with Venetoclax

Our analysis was largely based on conducting comparisons of AE frequency between reports using either drug (navitoclax or venetoclax) alone or in combination with any of the identified senescence-inducing chemotherapies. In order to determine which chemotherapy types should be included in the drug combination group, we reviewed the literature for evidence of TIS for all possible chemotherapies combined with either drug and reported by the FAERS. This review considered any chemotherapy drug to be a senescence inducer if any of the established markers of TIS were documented in a preclinical or clinical, hematological or non-hematological tumor cell model (Table 1). The markers of TIS include various established characteristic features of senescent cells. These included from morphological features (such as cellular enlargement and flattening), nuclear changes [27], increased granularity [28] and polyploidy [29]. The classical marker of senescence is increased activity of senescence-associated β-galactosidase (SA-β-gal), which is a component of broader senescence-associated lysosomal changes [27,30]. Others include Lamin B1 downregulation [31], formation of the senescence-associated heterochromatin foci (SAHF), and markers of activation of the DNA damage repair response (DDR) such as p53 phosphorylation and γH2AX foci formation [27]. Upregulation of cell cycle inhibitors such as p21^Cip1^, p16^INK4a^, p15^INK4b^, and p27^Kip1^ [27,32,33], as well as downregulation of proliferation markers like Ki67 [30], cyclin B1 [34,35], cyclin D1 [35], cyclin-dependent kinase (CDK) 4 and CDK2 [36], are also frequently utilized to identify the SAGA. Moreover, senescent cells are often identified by measuring Rb pathway activation [27] and amyloid β accumulation [37]. 

Our review identified a list of chemotherapeutic agents that were combined with either navitoclax or venetoclax and have previous evidence of TIS, whether in hematological or non-hematological cell lines (Table 1). TIS in hematological models was recorded with 17 agents from different drug classes involving topoisomerase poisons (e.g., doxorubicin, daunorubicin, and etoposide), CDK4/6 inhibitors (e.g., abemaciclib and palbociclib), monoclonal antibodies (e.g., rituximab and obinutuzumab), antimetabolites (e.g., cytarabine, actinomycin D, hydroxyurea, and decitabine), kinase inhibitors (e.g., imatinib and vemurafenib), alkylating agents (e.g., cyclophosphamide, melphalan, and cisplatin), and others (Table 1). These agents were associated with the expression of a wide range of senescence markers, for instance, SA-β-gal, formation of SAHF, cell cycle arrest, increased SASP production, upregulation of p21^Cip1^, p16^INK4^, and p27^Kip1^, and many other senescence hallmarks (Table 1).

TIS in non-hematological models was established with 34 agents used in combination with venetoclax or navitoclax, also from various drug classes; some are common with those drugs with evidence of senescence induction in hematological models, such as topoisomerase poisons (e.g., topotecan, irinotecan, and mitoxantrone), monoclonal antibodies (e.g., bevacizumab, trastuzumab, and ranibizumab), antimetabolites (e.g., methotrexate, gemcitabine, azacitidine, pemetrexed, 5-fluorouracil, and fludarabine), kinase inhibitors (e.g., erlotinib, nilotinib, sorafenib, dasatinib, trametinib, gefitinib, and ponatinib), alkylating agents (e.g., busulfan, temozolomide, and carmustine), and platinum-based agents (e.g., carboplatin and oxaliplatin). Other distinct drug classes include hormonal therapies (e.g., fulvestrant and tamoxifen), microtubule inhibitors (e.g., paclitaxel, vincristine, vinblastine, and docetaxel), the mTOR inhibitor rapamycin, the HDAC inhibitor panobinostat, and the PARP inhibitor olaparib (Table 1). Senescence markers that have been shown in these agents include SA-β-gal, SASP formation, DNA damage response, and upregulation of p16^INK4^, p21^Cip1^, p53, and p27^Kip1^ (Table 1). All drugs that were included in the drug combinations but did not have previous evidence in support of their ability to trigger TIS were excluded from the analysis. Those drugs were excluded after standard de-duplication procedures were applied, ensuring that case-level redundancy did not influence eligibility determination. Exclusion was performed to restrict the analytic dataset to regimens consistent with the definition of the “one–two punch” paradigm. 

### 2.3. Data Processing

FAERS adverse events are coded using the Medical Dictionary for Regulatory Activities (MedDRA); all hematological adverse effect groupings in this study were defined at the MedDRA preferred-term (PT) level using MedDRA v28.1 [92]. After de-duplication (described above), we performed data auditing/cleaning and then derived analysis variables using predefined formulas in Microsoft Excel, followed by statistical processing in R.

#### 2.3.1. Hematological Adverse-Effect Grouping (PT-Based)

**Figure 2:**
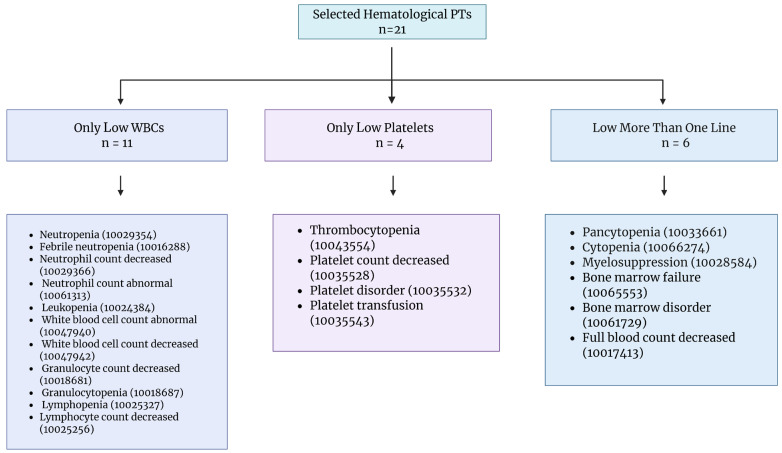
**Preferred-term (PT)-based framework used to classify hematologic adverse event reports in FAERS.** Schematic summary of the MedDRA preferred-term grouping rules used to derive the three hematologic adverse event categories analyzed in this study: isolated low white blood cell count, isolated low platelet count, and low counts affecting more than one blood cell line (multi-lineage cytopenia/bone marrow suppression).

Because a single FAERS report can list multiple PTs, we defined three hematological categories using PT membership rules: *(i)* only low WBC (isolated leukocyte-line abnormalities; reports were classified into this group when any of the following PTs were present and no platelet-line PTs and no multi-lineage PTs), *(ii)* only low platelet (isolated platelet-line abnormalities; reports were classified into this group when any of the following PTs were present and no WBC-line PTs and no multi-lineage PTs were present) and *(iii)* low more than one line (multi-lineage cytopenia/bone marrow suppression terms; reports were classified into this group when any of the following PTs were present) (Figure 2).

For disproportionality analyses, each hematologic category was evaluated as a separate binary endpoint; thus, for a given category, the comparator (“No”) group comprised all reports not meeting that specific category definition and was not restricted to reports without other hematologic PT groupings.

- Reports that did not meet criteria for any of the above categories were not considered to have the hematological adverse effect terms of interest for the hematology-specific analyses.

#### 2.3.2. Outcome Variable Derivation (FAERS Outcomes)

Patient outcomes were derived from the FAERS outcome fields as reported. We evaluated the following outcome categories as separate binary endpoints: death, life-threatening, hospitalization, or any of the other outcomes (disability, congenital anomaly, or required intervention). Because a single report may contain more than one outcome, outcome categories were not mutually exclusive (i.e., one report could contribute to multiple outcome endpoints). Thus, in the disproportionality tables, each outcome category (including death and life-threatening) was analyzed as an independent binary endpoint, and a single FAERS report could contribute to more than one outcome category if multiple outcomes were recorded.

### 2.4. Data Mining Algorithms (Disproportionality Analysis)

Disproportionality analysis was conducted using commonly applied frequentist pharmacovigilance metrics, namely the Reporting Odds Ratio (ROR) and the Proportional Reporting Ratio (PRR), which are derived from a 2 × 2 contingency table [93,94,95]. These measures are widely used for exploratory signal detection in spontaneous reporting systems; however, they do not establish causality and should not be interpreted as incidence or risk estimates [96]. For the main comparative analysis, we constructed 2 × 2 tables comparing venetoclax monotherapy vs. venetoclax combined with senescence-inducing therapy for each hematological AE category and for each outcome category:

a = Number of reports in the combination group with the AE (or outcome) of interest;

b = Number of reports in the combination group without the AE (or outcome) of interest;

c = Number of reports in the monotherapy group with the AE (or outcome) of interest;

d = Number of reports in the monotherapy group without the AE (or outcome) of interest.

The ROR was calculated as (a/b)/(c/d) = (a × d)/(b × c). The PRR was calculated as [a/(a + b)]/[c/(c + d)]. For each measure, 95% confidence intervals (CIs) were computed using standard log-transformed methods. Statistical significance was assessed using Pearson’s chi-squared test with Yates’ continuity correction for 2 × 2 tables, and *p* < 0.05 was considered statistically significant. No pre-specified minimum cell count threshold was applied for the venetoclax disproportionality analyses because the analyzed venetoclax comparisons involved large report counts. Navitoclax was not subjected to formal disproportionality analysis and was instead summarized descriptively because of the limited number of reports. In interpreting disproportionality findings, we prioritized the direction and magnitude of the ROR/PRR estimates and their 95% confidence intervals, while chi-squared p-values were used as supportive statistical descriptors. We selected ROR and PRR as transparent, commonly used frequentist signal detection metrics for this predefined monotherapy versus combination comparison. Bayesian shrinkage methods (e.g., IC or EBGM) were not applied in the current study; although they may be valuable, particularly in sparse data settings, their use was beyond the scope of this analysis. Because navitoclax had a limited number of total reports, analyses for navitoclax were restricted to descriptive reporting rather than formal disproportionality comparisons.

### 2.5. Ethics and Reporting Considerations

FAERS is a publicly available, de-identified spontaneous reporting database. This study analyzed aggregated FAERS case reports and did not involve direct contact with human participants, access to identifiable private information, or any intervention. Therefore, institutional review board (IRB) approval and informed consent were not required. Because FAERS is a spontaneous reporting system, reported drug–event associations represent suspected relationships and are subject to under-reporting, missing data, reporting bias, duplicate submissions, and confounding (including confounding by indication and co-medication). Accordingly, the results should be interpreted as signal detection/disproportionality in reporting and not as incidence or causal risk estimates.

### 2.6. Software and Statistical Analysis

All data cleaning, auditing, and derivation of analysis variables (including the hematological PT-based groupings and outcome indicators) were performed using Microsoft Excel (Microsoft 365). Disproportionality metrics (ROR and PRR) and their 95% confidence intervals were calculated from 2 × 2 contingency tables as described above, and Pearson’s chi-squared test with Yates’ continuity correction was used to evaluate differences between groups. The indication- and sex-stratified analyses were performed as descriptive subgroup summaries only; no formal interaction testing was pre-specified or conducted. For descriptive summaries, percentages were calculated within the relevant comparison stratum (i.e., column-based percentages unless otherwise specified). Statistical analyses were performed in R (RStudio 2023.09.1+494). All statistical tests were two-sided, and *p* < 0.05 was considered statistically significant.

## 3. Results

### 3.1. Descriptive Analysis

A total of 47,508 venetoclax FAERS reports met the final analytic inclusion criteria and were included in the present analysis. Of these reports, 72.6% (*n* = 34,485) involved venetoclax monotherapy, while 27.4% (*n* = 13,023) involved venetoclax combined with a senescence-inducing therapy. Only low white blood cell (WBC) count was reported in 8.3% of records (*n* = 3939), only a low platelet count in 2.9% (*n* = 1384), and a low level of more than one line of blood cells in 12.3% (*n* = 5867) (Table 2). Regarding outcomes, life-threatening outcomes were reported in 3.2% of records (*n* = 1509), hospitalization in 37.7% (*n* = 17,918), non-serious outcomes in 9.8% (*n* = 4671), other outcomes in 42.6% (*n* = 20,233), and death in 30.7% (*n* = 14,580). It is noteworthy that the proportion of each outcome does not reflect drug-attributable effects exclusively and could also indicate advanced disease severity, as FAERS spontaneous reports do not permit adjudication of causality.

### 3.2. Signal Detection of Venetoclax Monotherapy and Combination with Senescence-Inducing Therapy

To assess the difference in adverse hematological outcomes between venetoclax monotherapy and combination with senescence-inducing therapy, disproportionality analyses were performed for three categories of adverse events: only low WBC count, only low platelet count, and low counts in more than one blood cell line (multi-lineage cytopenia/pancytopenia). Reports were grouped into these three categories to capture the spectrum and severity of marrow toxicity and to differentiate lineage-specific effects from global marrow suppression, thereby improving the specificity of signal detection. For isolated low WBC count, combination therapy showed a higher reporting frequency than venetoclax monotherapy (15% vs. 5.8%), despite far fewer total reports in the combination group (Table 2). Disproportionality analysis demonstrated an elevated signal, with a Reporting Odds Ratio (ROR) of 2.87 (95% CI: 2.69–3.07) and a Proportional Reporting Ratio (PRR) of 2.59 (95% CI: 2.44–2.75), supported by a chi-squared value of 1047.5 (*p* < 2.2 × 10^−16^) (Table 3). Overall, low WBC count was disproportionately reported in the combination group compared with monotherapy. In contrast, isolated low platelet count was markedly less frequent in the combination group compared with monotherapy (1.1% vs. 3.6%) (Table 2). The disproportionality signal indicated a lower association with isolated thrombocytopenia for combination therapy, with an ROR of 0.31 (95% CI: 0.26–0.37), a PRR of 0.32 (95% CI: 0.27–0.38), and a chi-squared value of 195.9 (*p* < 2.2 × 10^−16^) (Table 3). 

For low counts affecting more than one blood cell line, reports were more frequent in the combination group compared with monotherapy (23.7% vs. 8.1%) (Table 2). Disproportionality analysis showed a strong signal, with an ROR of 3.54 (95% CI: 3.35–3.75) and a PRR of 2.94 (95% CI: 2.81–3.08), supported by a chi-squared value of 2135.5 (*p* < 2.2 × 10^−16^) (Table 3), indicating that combination therapy is disproportionately linked to multi-lineage cytopenias compared with venetoclax monotherapy.

These results indicate a marked difference in hematological AE profiles between venetoclax monotherapy and combination with senescence-inducing therapy, with disproportionately higher reporting of low WBC and multi-lineage cytopenias in the combination group, suggesting a stronger reporting signal consistent with greater marrow suppression when venetoclax is combined with senescence-inducing therapy. Conversely, an isolated low platelet count was disproportionately less frequently reported in the combination group than with monotherapy.

### 3.3. Outcome Occurrence with Venetoclax Monotherapy and Combination with Senescence-Inducing Therapy

A disproportionality analysis was conducted to evaluate the occurrence of five outcome categories following the use of venetoclax monotherapy or in combination with senescence-inducing therapy. Both ROR and PRR, along with their 95% CIs, were calculated for each outcome category. All associations demonstrated statistically significant differences, with *p* < 0.001 for all comparisons. Since FAERS outcome fields are non-mutually exclusive, these outcome-specific ROR/PRR estimates should be interpreted as separate reporting signals for each endpoint, and overlap between outcomes (e.g., death and hospitalization in the same report) may contribute to correlated reporting patterns across categories.

Overall, combination therapy showed higher disproportionality reporting signals for life-threatening outcomes, hospitalization, and other outcomes, while death and non-serious outcomes were proportionally less reported compared with monotherapy (all *p* < 0.001; Table 4). The strongest positive signal was observed for life-threatening outcomes (ROR 7.06; PRR 6.56), whereas death (ROR 0.55; PRR 0.65) and non-serious outcomes (ROR 0.26; PRR 0.29) showed inverse disproportionality (Table 4). Detailed ROR, PRR, 95% CI, and chi-squared values for each outcome are provided in Table 4. It is worth mentioning that the observed pattern of lower reported death alongside higher life-threatening and hospitalization signals may reflect differential reporting patterns within the FAERS, including earlier clinical intervention prompted by severe deficiencies of blood components or potential increased reporting sensitivity for acute but reversible complications. 

### 3.4. Exploratory Outcome Pattern Analyses (Overlap, Indication, and Sex Stratification) 

Because the FAERS outcome fields are not mutually exclusive, we examined overlap patterns among death, life-threatening, and hospitalization outcomes. In venetoclax reports, the proportion with none of these three outcomes was similar between monotherapy and combination (36.3% vs. 36.9%, respectively). However, hospitalization only was more frequent in the combination group (35.7% vs. 29.0%), while death only was less frequent (13.2% vs. 29.2%). Overlap patterns involving life-threatening outcomes were also more frequent with combination therapy, including life-threatening + hospitalization (3.56% vs. 0.44%) and death + life-threatening + hospitalization (2.18% vs. 0.35%) (Table S1). These stratified analyses (by indication and sex) were descriptive and intended to contextualize reporting patterns; no formal interaction testing was conducted.

**Figure 3:**
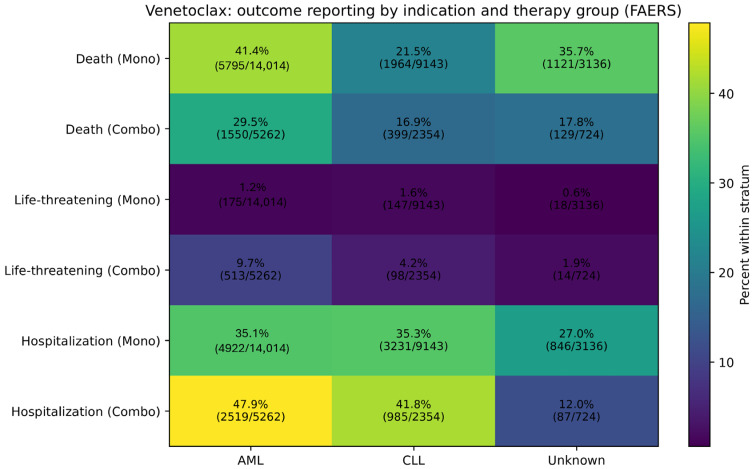
Venetoclax outcome reporting by indication and therapy group (FAERS). Heatmap showing the proportion of reports with death, life-threatening, and hospitalization outcomes within each stratum defined by the primary “Reason for Use” category (AML, CLL, and unknown/unspecified) and therapy group (venetoclax monotherapy vs. venetoclax + senescence-inducing therapy). Values are displayed as percent within stratum, with counts (n/N) in each cell. Outcomes are not mutually exclusive.

To explore whether outcome patterns varied across major indications, we performed an indication-stratified descriptive analysis for venetoclax using the primary “Reason for Use”. In AML, the combination group showed a lower proportion of death reports (29.5% vs. 41.4% in monotherapy), while life-threatening (9.7% vs. 1.2%) and hospitalization (47.9% vs. 35.1%) were proportionally more frequent. Similar directional patterns were observed in CLL, with lower death (16.9% vs. 21.5%) and higher life-threatening (4.2% vs. 1.6%) and hospitalization (41.8% vs. 35.3%) outcomes in the combination group (Figure 3; Table S2). These indication-stratified analyses were descriptive and are presented to contextualize reporting patterns; differences across strata may reflect underlying differences in baseline prognosis, disease severity, and treatment setting rather than treatment effects alone.

Finally, outcomes were summarized by sex. In the venetoclax combination group, life-threatening outcome proportions were similar in males and females (9.3% vs. 9.4%), with comparable hospitalization proportions (54.6% vs. 52.6%); death was modestly higher in males (22.1%) than in females (20.2%). In monotherapy, life-threatening proportions were similarly low in males and females (~1.29%), while death was higher among male reports (35.7%) than female reports (31.6%) (Table S3).

For navitoclax (descriptive only due to limited reports), outcome overlap patterns similarly showed substantial co-reporting, with hospitalization-only (45.9%) and life-threatening + hospitalization (14.5%) outcomes among the most frequent patterns (Table S4). Sex-stratified summaries and regimen-stratified descriptive outcomes (based on co-suspect partners) are provided in Tables S5 and S6. 

## 4. Discussion

Since senolytics are proposed for use in the “one-two punch” approach, in which they are combined with senescence-inducing therapies, a real-world revision of the expected AEs and outcomes in combination settings is crucial. It is noteworthy that previous studies have conducted similar investigations of the AE profile of venetoclax using the FAERS database [97,98]. However, the present study represents a unique FAERS-based effort framed within the senotherapeutic “one-two punch” approach strategy and includes a large number of reports, particularly for venetoclax (47,508 cases) and over a long period. Also, to our knowledge, it is the first study that executed a disproportionality analysis comparing venetoclax monotherapy to venetoclax in combination with senescence-inducing chemotherapy. It must be clearly stressed that although this study is framed within the conceptual senolytic therapy paradigm, the FAERS does not document treatment sequencing, and accordingly, our findings should be interpreted as hypothesis-generating safety signals derived from real-world co-exposure to venetoclax and senescence-inducing therapies.

The present study included all available reports on venetoclax and navitoclax until Q2 2025, derived from the FAERS database. Descriptive analysis was performed for both venetoclax and navitoclax and demonstrated that the reported hematological AEs included mainly multi-lineage cytopenias, low WBC count, and low platelet count. In the outcome analysis, hospitalization, death, and other outcomes were the most commonly reported outcomes. To a lesser extent, life-threatening and non-serious events were also reported. For venetoclax, when combined with senescence-inducing chemotherapy, hematological AEs showed disproportionate reporting signals for low WBC counts and multi-lineage cytopenias, whereas low platelet count was reported significantly less frequently. In addition, based on the outcome analysis, the combination arm showed higher reporting signals for life-threatening events, hospitalizations, and other outcomes, while death and non-serious outcomes were proportionally less reported. As with all FAERS-based analyses, these findings reflect reporting patterns (signal strength) rather than incidence or true event rates.

Two previous studies have been conducted using the FAERS database on venetoclax [97,98]. The first analysis, by Yang et al. in 2022, included 19,107 reports on venetoclax between 2016 and 2021 [97]. Disproportionality analysis was performed at the MedDRA system organ class (SOC) and PT levels. They reported statistically significant signals for hematological AEs, including cytopenia (ROR 14.57, PRR 14.47), neutropenia (ROR 7.05, PRR 6.81), and WBC count decreased (ROR 7.71, PRR 7.51). In addition, positive signals were reported for thrombocytopenia (ROR 4.07, PRR 4.01) and platelet count decreased (ROR 10.70, PRR 10.23). Moreover, they also reported an overall SOC-level signal for blood and lymphatic system disorders (ROR 5.91, PRR 4.77). This analysis demonstrates a strong and consistent pharmacovigilance signal linking venetoclax to hematological AEs, particularly cytopenias, neutropenia, and thrombocytopenia. Importantly, in this work and ours, it must be highlighted that alternative explanations for the observed increase in cytopenia signals should be considered, independent of the mere combination of venetoclax and other forms of chemotherapy (particularly bone marrow suppressive), including greater cumulative dose intensity in combination regimens, polypharmacy-related marrow suppression, more advanced disease severity in patients selected for multi-agent therapy, and differential reporting behavior in the setting of complex treatment protocols. 

In the second study by Kt et al., a meta-analysis of randomized controlled trials (RCTs) and a retrospective evaluation for venetoclax-associated AEs based on the FAERS database was conducted. It included seven studies with a total of 1730 patients, of whom 978 were within the venetoclax treatment group. Their results showed that hematological AEs were predominant, such as neutropenia, febrile neutropenia, and thrombocytopenia, all of which were reported with venetoclax both as monotherapy and when combined with other chemotherapies, such as hypomethylating agents. Disproportionality analysis (including available data until the third quarter of 2023) also reported signals for hematological AEs, including neutropenia (ROR 7.30, PRR 7.49), thrombocytopenia (ROR 6.15, PRR 6.47), pancytopenia (ROR 8.01, PRR 8.63), and WBC count decreased (ROR 5.44, PRR 5.75), with a modest reduction in ratios when they restricted the analyses to primary suspect reports. It is noteworthy that the control group was not clearly defined as to which the venetoclax treatment group was compared to in that FAERS-based disproportionality analysis. Accordingly, we could not fully conclude whether the signals for hematological AEs directly support our specific monotherapy versus combination comparison. However, the persistence of significant disproportionality signals, even after restriction to primary suspect reports, is consistent with a robust pharmacovigilance signal for venetoclax-associated bone marrow suppression across settings. Our PT-based grouping was intended to prioritize robust signal detection in FAERS rather than detailed clinical phenotyping of marrow toxicity combinations.

In support of these findings, another study that was conducted specifically based on the French national pharmacovigilance database (FNPVD) for venetoclax included 123 patients and showed that the most commonly reported AEs were the hematological, accounting for 21% of all studied AEs, more specifically neutropenia and febrile neutropenia (10%), followed by thrombocytopenia (3%) [99]. Our results align with these previous studies’ results collectively in regard to the hematological AEs, with positive signals for multi-lineage cytopenia (ROR 3.54, PRR 2.94) and low WBC count (ROR 2.87, PRR 2.59). However, we report an inverse disproportionality signal for low platelet count (ROR 0.31, PRR 0.32). This observation may reflect differential hematologic reporting patterns in the combination setting, including possible coding shifts toward broader marrow-suppression or multi-lineage PTs (e.g., pancytopenia/cytopenia/myelosuppression) rather than isolated platelet-line terms within our PT-based grouping scheme. Importantly, the elevated odds ratio for multi-lineage cytopenias (ROR 3.54) in our reporting can be interpreted in the context of expected additive or synergistic marrow suppression from concomitant cytotoxic or myelosuppressive effects of senescence-inducing chemotherapy, as combination regimens in hematologic malignancies inherently carry this cumulative risk beyond a potential potentiation based on the “one-two punch” approach. Still, this increased risk for cytopenias continues to represent a potential challenge for this therapeutic strategy.

Evidence from prospective RCTs further delineates the hematologic safety profile of venetoclax across both monotherapy and combination treatment settings. For example, a phase 3b trial assessing AEs associated with venetoclax monotherapy revealed that neutropenia (37%), anemia, and thrombocytopenia (both 13%) were the most frequently reported grade 3/4 AEs [100]. Other clinical trials measured the AEs of venetoclax in combination with chemotherapies such as LDAC, which showed frequent reporting of neutropenia, febrile neutropenia, and thrombocytopenia [101,102].

For navitoclax, many clinical trials were also executed to evaluate its associated toxicity. In a phase 2a trial, navitoclax, as monotherapy, was reported to be associated primarily with grade 3/4 thrombocytopenia (38.5%), followed by neutropenia (30.8%) and other non-hematological AEs, such as diarrhea (88.5%) and nausea (61.5%) [9]. Another trial studied the AEs of navitoclax when combined with venetoclax and chemotherapy backbone and documented frequent hematological grade 3/4 adverse events, including febrile neutropenia (47%), neutropenia (38%), and thrombocytopenia (26%), which is at a lower frequency than thrombocytopenia recorded with navitoclax monotherapy in an earlier trial [103], potentially attributable to the use of low-dose navitoclax [104].

An important limitation of our analysis was that it was only descriptive for navitoclax, due to the limited number of reports (172 reports). This is because it did not gain FDA approval as an anticancer agent; therefore, these limited reports are likely to represent the reports related to navitoclax clinical trials. One more limitation is that we included all reports where venetoclax was reported either as a primary or secondary suspect, making our analysis less specific. Moreover, the disease itself, hematological malignancies, may serve as a confounding factor that contributes to cytopenia. In addition, the exploratory indication-stratified outcome patterns (e.g., AML vs. CLL) may be influenced by differences in baseline prognosis, disease severity, and clinical treatment context across indications. Therefore, these findings should be interpreted as descriptive reporting patterns and not as evidence of causal differences in outcomes attributable to the treatment regimen. Furthermore, the multi-lineage cytopenia category was defined by the presence of multi-lineage/bone marrow suppression PTs regardless of co-listed isolated lineage PTs. Although this approach was chosen to reflect clinically relevant bone marrow suppression coding patterns in the FAERS, we did not perform a sensitivity analysis excluding reports with overlapping PT coding, which may affect the distribution of PT-based hematologic categories. Moreover, the multi-lineage cytopenia category was analyzed as a broad PT-based pharmacovigilance grouping and was not further decomposed into specific lineage combinations (e.g., neutropenia + thrombocytopenia vs. anemia + neutropenia). Such characterization may provide greater clinical relevance particularly for agents with known lineage-specific toxicity profiles (e.g., Bcl-xL-dependent platelet toxicity with navitoclax) and should be explored in future studies. 

Finally, an additional important limitation of this work is that the classification of chemotherapy agents as “senescence-inducing” was based exclusively on prior preclinical evidence in various cancer models, including both hematological and non-hematological malignancies. Of course, none of the clinical FAERS reports provide direct or indirect evidence confirming senescence induction in patients receiving those drugs, and it is certainly possible that some of these agents may not be effectively inducing senescence in hematological tumor cells in the clinical setting [105]. Moreover, the proposed “one-two punch” strategy suggests, in part, a sequential therapeutic pattern where senescence induction is followed by senolytic culling of tumor cells [24], whereas in the analyzed reports in this study, drug administration might involve overlapping schedules. This difference is also associated with different toxicity patterns and limits direct extrapolation of our findings to optimized senotherapeutic regimens. Lastly, the exclusion of chemotherapy that is not known to induce senescence from the analysis may have introduced selection bias and enriched for regimens with distinct hematologic toxicity profiles. However, this restriction was driven by the original hypothesis and was necessary to isolate safety signals specifically attributable to the “one-two punch” strategy.

It is noteworthy that our FAERS-based analysis relies on voluntary reporting and is therefore affected by under-reporting, reporting bias, and variable report quality. In addition, the absence of reliable exposure denominators prevents estimation of true incidence rates or risks; consequently, disproportionality metrics such as ROR and PRR represent reporting signals rather than measures of causal association. Although standard de-duplication procedures were applied, residual duplicate reports cannot be completely excluded. FAERS reports also frequently lack detailed clinical information, including comorbidities, disease severity, treatment duration, and concomitant therapies, which may introduce confounding. Therefore, the present analysis should be interpreted primarily as signal detection rather than establishing causal relationships.

Taken together, previous analyses, as well as ours, indicate that the use of venetoclax in combination with chemotherapy, including senescence-inducing regimens, is associated with a potentially less favorable AE profile compared to its use as a monotherapy, characterized by disproportionately higher reporting of leukopenia and multi-lineage cytopenias and higher reporting signals for serious clinical outcomes such as hospitalizations and life-threatening events. These observations propose some important translational concerns for the clinical implementation of the senotherapeutic “one-two punch” strategy, as the potential myelosuppressive burden imposed by combining senescence induction with senolytic clearance may narrow the therapeutic window and limit tolerability. Consequently, successful translation of current senolytics into cancer treatment will require further understanding of their hematologic risk. This pharmacovigilant challenge represents an additional barrier against the senotherapeutic strategy for cancer treatment to the current preclinical unresolved issues associated with their investigation, including variable selectivity, differential effects across different cancer models, and poorly developed in vivo efficacy [11,12]. Addressing these challenges is essential before senolytics can be reliably integrated into cancer therapy. Lastly, it is important to emphasize that venetoclax-based combinations remain established standards of care in various leukemia types, with well-documented survival benefits. Accordingly, the detected pharmacovigilance signals should not be interpreted as negating proven clinical efficacy but rather as informing risk stratification and safety monitoring within evolving translational applications. Moreover, given the inherent limitations of spontaneous reporting systems, these findings should be interpreted as pharmacovigilance signals that warrant further evaluation in controlled clinical or real-world observational studies.

## Figures and Tables

**Table 1:** Chemotherapeutic agents identified in the FAERS-reported drugs in combination with venetoclax or navitoclax and associated with TIS. Summary of drugs that were combined with venetoclax or navitoclax in FAERS-derived reports that demonstrated senescence induction among either hematological or non-hematological models.

Drug Class	Drug Name	Type of Malignancy	Model	Senescence Hallmarks	Reference
**Evidence of TIS in hematological tumor models**					
Topoisomerase poisons	Doxorubicin (Adriamycin)	Hematological	CML (K562 cells), Burkitt’s lymphoma (Raji cells), and DLBCL (SU-DHL-2)	SA-β-gal, enlarged cell size, SAHF formation, and G1 cell cycle arrest	[38,39]
	Daunorubicin	Hematological	AML (OCI-AML5, OCIM2, and murine myeloid leukemia cell lines)	SA-β-gal, enlarged cell size, G2/M cell cycle arrest, and upregulation of SASP	[40]
	Etoposide	Hematological	Burkitt’s lymphoma (Raji-4RH) and DLBCL (RL-4RH)	SA-β-gal, increased p53 expression, p21^Cip1^ upregulation, G2/M cell cycle arrest, and DNA damage response activation	[41]
CDK 4/6 inhibitors	Abemaciclib	Hematological	DLBCL (OCI-LY3 and A20)	SA-β-gal, p53 and p21^Cip1^ upregulation, and Ki67 downregulation	[42]
	Palbociclib	Hematological	AML (AML blasts)	SA-β-gal and G1 cell cycle arrest	[43]
Monoclonal antibodies	Rituximab	Hematological	CLL (EHEB), DLBCL (RC-K8), ALL (SD-1), and EBV-transformed primary human B-lymphoblastoid cells	SA-β-gal and morphological changes	[44]
	Obinutuzumab	Hematological	Follicular lymphoma 3D model	SA-β-gal	[45]
Antimetabolite	Cytarabine (Ara-C)	Hematological	AML (KG-1 AML cell line and patient-derived AML cells)	SA-β-gal, enlarged cell size, increased granularity, SASP activation, p21^Cip1^ upregulation, and DNA damage response activation	[46]
	Actinomycin D	Hematological	AML (AML2 and AML3 cells)	SA-β-gal, increased p53 expression, p21^Cip1^ upregulation, and reduced clonogenic potential	[36]
	Hydroxyurea	Hematological	CML (K562)	SA-β-gal and increased expression of p16^INK4a^, p21^Cip1^, and p27^Kip1^	[32]
	Decitabine (5-aza-2’-deoxycytidine)	Hematological	CML (k-562, MEG-01, KBM-5)	SA-β-gal, p16/p27 mRNA/protein, p21 mRNA, morphology, and downregulation of Htert	[33]
Kinase inhibitors	Imatinib	Hematological	CML (K562)	SA-β-gal, cell cycle arrest, p21^Cip1^, and p27^Kip1^ upregulation	[47]
	Vemurafenib	Hematological	AML (SKM-1, MOLM-13 cells)	Lamin B1 and TRF2 downregulation, G0/G1 cell cycle arrest, p16^INK4a^ and p21^Cip1^, and decreased expression of CDK4 and CDK2.	[48]
Alkylating agents	Cyclophosphamide	Hematological	HSC-bcl2 lymphoma	SA-β-gal, p53 activation, and p16^INK4a^ upregulation	[49]
	Melphalan	Hematological	Multiple myeloma (5TGM1 cells)	SA-β-gal	[50]
Platinum-coordination complexes	Cisplatin	Hematological	Follicular lymphoma 3D model	SA-β-gal	[45]
Selective inhibitor of nuclear export (SINE)	Selinexor	Hematological	DLBCL cells	SA-β-gal and p53 upregulation	[51]
**Evidence of TIS in non-hematological tumor models**					
Topoisomerase poisons	Topotecan	Non-hematological	Neuroblastoma	SA-β-gal, reduced DNA synthesis, morphology, growth arrest, and p21^Cip1^	[52]
	Irinotecan	Non-hematological	Gastric adenocarcinoma	SA-β-gal	[53]
	Mitoxantrone	Non-hematological	Lung cancer	SA-β-gal, growth arrest, γH2AX formation, and morphological changes	[54,55]
Monoclonal antibodies	Bevacizumab	Non-hematological	Colorectal adenocarcinoma	SA-β-gal	[56]
	Trastuzumab	Non-hematological	Breast cancer	SA-β-gal, p15^INK4^ and p16^INK4a^ upregulation	[57]
	Ranibizumab	Non-hematological	Primary porcine retinal pigment epithelial cells	SA-β-gal, cathepsin D expression, and amyloid β accumulation	[37]
Antimetabolite	Methotrexate	Non-hematological	Neuroblastoma and colon, lung, and breast cancers	SA-β-gal, p21^Cip1^ upregulation, Ki67 downregulation, growth arrest, increased granularity, morphological changes, γH2AX foci formation, and SASP activation	[58,59]
	Gemcitabine	Non-hematological	Pancreatic adenocarcinoma	SA-β-gal	[60]
	Azacitidine	Non-hematological	Breast and prostate cancers, papillary thyroid carcinoma, cholangiocarcinoma, and osteosarcoma	SA-β-gal, SASP activation, growth arrest, morphological changes, and polyploidy	[61,62,63,64]
	Pemetrexed	Non-hematological	Lung cancer	SA-β-gal, morphological changes and SASP activation	[65]
	5-Fluorouracil	Non-hematological	Hepatocellular carcinoma and breast cancer	SA-β-gal	[66,67]
	Fludarabine	Non-hematological	Breast cancer	SA-β-gal, Lamin B1 downregulation, and p21^Cip1^ upregulation	[68]
Tyrosine kinase inhibitors (TKIs)	Erlotinib	Non-hematological	Lung cancer	SA-β-gal and morphological changes	[69]
	Nilotinib	Non-hematological	Lung cancer	SA-β-gal	[70]
	Sorafenib	Non-hematological	Hepatocellular carcinoma	SA-β-gal, p16^INK4a^ upregulation, and SASP activation	[71]
	Dasatinib	Non-hematological	Lung cancer	SA-β-gal, p21^Cip1^ upregulation, and γH2AX foci formation	[72]
	Trametinib	Non-hematological	Melanoma and lung cancer	SA-β-gal, growth arrest, p53 activation, p21^Cip1^ upregulation, and SASP activation	[73,74,75]
	Gefitinib	Non-hematological	Lung cancer and esophageal squamous cell carcinoma	Growth arrest, p16^INK4^, p21^Cip1^, p53, and p27^Kip1^ upregulation	[76]
	Ponatinib	Non-hematological	Primary human aortic endothelial cells (HAECs)	SA-β-gal	[77]
Alkylating agents	Busulfan	Non-hematological	Osteosarcoma	SA-β-gal	[78]
	Dacarbazine	Non-hematological	Melanoma	SASP activation	[79]
	Temozolomide	Non-hematological	Glioblastoma	SA-β-gal and morphological changes	[80]
	Carmustine	Non-hematological	Glioma	SAHF formation and p53 and Rb activation	[35]
Platinum based	Carboplatin	Non-hematological	Lung cancer	SA-β-gal, cell cycle arrest, p16^INK4a^ and Rb activation, and cyclin B1 and cyclin D1 downregulation	[81]
	Oxaliplatin	Non-hematological	Hepatocellular carcinoma	SA-β-gal	[82]
Hormonal therapy	Fulvestrant	Non-hematological	Breast cancer	SA-β-gal	[83]
	Tamoxifen	Non-hematological	Breast and colorectal cancers	SA-β-gal and p53 and p21^Cip1^ upregulation	[84]
Microtubule inhibitors	Paclitaxel	Non-hematological	Neuroblastoma and lung, breast, and colorectal cancers	SA-β-gal, growth inhibition, γH2AX foci formation, morphological changes, SASP activation, p21^Cip1^ upregulation, Ki67 downregulation, and increased granularity	[59]
	Vincristine	Non-hematological	Breast cancer	Senescence-associated lysosomal changes and morphological changes	[85]
	Vinblastine	Non-hematological	Patient-derived glioma cells	Nuclear morphometric changes and cell cycle arrest	[86]
	Docetaxel	Non-hematological	Ovarian cancer	SA-β-gal, γH2AX foci formation, polyploidy, and G2/M cell cycle arrest	[87]
mTOR inhibitors	Rapamycin (Sirolimus)	Non-hematological	Hepatocellular carcinoma	SA-β-gal	[66]
Histone deacetylase (HDAC) inhibitors	Panobinostat	Non-hematological	Lung and breast cancers	SA-β-gal, morphology, SASP, cyclinA2 downregulation, and p21^Cip1^ upregulation	[88,89]
PARP inhibitors	Olaparib	Non-hematological	Ovarian, breast, and colorectal cancers	SA-β-gal, growth arrest, γH2AX and 53BP1 foci formation, p21^Cip1^, p27^Kip1^, p15^INK4^, p16 ^INK4^, and p57 upregulation, and SASP activation	[90,91]

Abbreviations: SA-β-gal, senescence-associated β-galactosidase; SAHF, senescence-associated heterochromatin foci; SASP, senescence-associated secretory phenotype; DDR, DNA damage repair response; TRF2, telomeric repeat-binding factor 2; CDK, cyclin-dependent kinase; hTERT, human telomerase reverse transcriptase; Rb, retinoblastoma protein; 53BP1, p53-binding protein 1.

**Table 2:** Frequency reporting of hematologic adverse events with venetoclax monotherapy versus combination with senescence-inducing therapy. Raw counts of hematologic adverse event (AE) reports associated with venetoclax used as monotherapy or in combination with senescence-inducing therapy. Events were categorized into: isolated low white blood cell (WBC) count, isolated low platelet count, and low counts in more than one blood cell lineage (multi-lineage cytopenia/pancytopenia). For each event category, the number of reports that met the event definition and those that did not are shown for each treatment group. For each hematologic category, “No” indicates reports that did not meet that specific PT-based endpoint definition; therefore, “No” groups may include reports classified under other hematologic categories (e.g., isolated low WBC, isolated low platelet, or multi-lineage cytopenia), as well as reports without the predefined hematologic PT groupings. Percentages are column-based and were calculated within each treatment group (venetoclax monotherapy vs. combination with senescence-inducing therapy).

**Only Low WBC Count**		
	Low WBC (Yes)	Low WBC (No)
Combination	1948/13,023 (15%)	11,075
Monotherapy	1991/34,485 (5.8%)	32,494
Total	3939/47,508 (8.3%)	43,569
**Only Low Platelet Count**		
	Low Platelets (Yes)	Low Platelets (No)
Combination	150/13,023 (1.1%)	12,873
Monotherapy	1234/34,485 (3.6%)	33,251
Total	1384/47,508 (2.9%)	46,124
**Low Counts in More Than One Line of Blood Cells**		
	Multi-line Cytopenia (Yes)	No Cytopenia
Combination	3087/13,023 (23.7%)	9936
Monotherapy	2780/34,485 (8.1%)	31,705
Total	5867/47,508 (12.3%)	41,641

**Table 3:** Disproportionality analysis of hematologic adverse events associated with monotherapy versus combination with senescence-inducing therapy. Disproportionality metrics evaluating the association between venetoclax (monotherapy vs. combination with senescence-inducing therapy) and three hematologic adverse event (AE) categories. The Reporting Odds Ratio (ROR) and Proportional Reporting Ratio (PRR), each with 95% confidence intervals, quantify the strength of association between treatment type and AE reporting. Statistical significance was assessed using Pearson’s chi-squared test with Yates’ correction.

Disproportionality Analysis of Hematological Adverse Events			
Adverse Event	ROR (95% CI)	PRR (95% CI)	*Chi*-Squared (*p*-Value)
Only Low WBC	2.87 (2.69–3.07)	2.59 (2.44–2.75)	1047.5 (*p* < 2.2 × 10^−16^)
Only Low Platelets	0.31 (0.26–0.37)	0.32 (0.27–0.38)	195.9 (*p* < 2.2 × 10^−16^)
Multi-line Cytopenia	3.54 (3.35–3.75)	2.94 (2.81–3.08)	2135.5 (*p* < 2.2 × 10^−16^)

**Table 4:** Disproportionality analysis of outcomes associated with monotherapy versus combination with senescence-inducing therapy. The analysis employed ROR and PRR to assess signal detection. Significant positive disproportionality (lower limit of 95% confidence interval [CI] > 1.0) was detected for *life-threatening* outcomes (ROR 7.06, 95% CI: 6.3–7.91; PRR 6.56, 95% CI: 5.88–7.32), other outcomes (ROR 2.36, 95% CI: 2.27–2.46; PRR 1.57, 95% CI: 1.54–1.61), and *hospitalization* (ROR 1.74, 95% CI: 1.67–1.82; PRR 1.39, 95% CI: 1.36–1.42). Conversely, a statistically significant negative disproportionality (ROR/PRR < 1.0 with upper limit of 95% CI < 1.0) was observed for *death* (ROR 0.55, PRR 0.65) and *non-serious outcomes* (ROR 0.26, PRR 0.29). All detected signals were highly statistically significant, as indicated by a Pearson chi-square test (all *p* < 0.001, *df* = 1).

	ROR	CI (ROR)	Pearson Chi Square	Df	*p*-Value	PRR	CI (PRR)
**Death**	0.55	0.52–0.57	642.02	1	*p* < 0.001	0.65	0.62–0.67
**Life threatening**	7.06	6.3–7.91	1502.2	1	*p* < 0.001	6.56	5.88–7.32
**Hospitalization**	1.74	1.67–1.82	714.68	1	*p* < 0.001	1.39	1.36- 1.42
**Non-serious outcomes**	0.26	0.24–0.29	806.1	1	*p* < 0.001	0.29	0.26–0.32
**Other outcomes**	2.36	2.27–2.46	1720.5	1	*p* < 0.001	1.57	1.54–1.61

## Data Availability

The datasets analyzed as part of the current study are publicly available from the U.S. FDA Adverse Event Reporting System (FAERS) database.

## Supplementary Materials

The following supporting information can be downloaded at https://www.mdpi.com/article/10.3390/jcm15062194/s1, Table S1: Venetoclax outcome overlap patterns by therapy group (FAERS). Table S2: Venetoclax indication-stratified outcomes by therapy group (FAERS). Table S3: Venetoclax sex-stratified outcomes by therapy group (FAERS). Table S4: Navitoclax outcome overlap patterns (FAERS; descriptive). Table S5: Navitoclax sex-stratified outcomes (FAERS; descriptive). Table S6: Navitoclax regimen-stratified outcomes by co-suspect partner (FAERS; descriptive).

## Abbreviations

The following abbreviations are used in this manuscript:

53BP1	p53-binding Protein 1
AE	Adverse Event
ALL	Acute Lymphoblastic Leukemia
AML	Acute Myeloid Leukemia
BH3	Bcl-2 Homology 3
CDK	Cyclin-dependent Kinase
CDKI	Cyclin-dependent Kinase Inhibitor
CI	Confidence Interval
CLL	Chronic Lymphocytic Leukemia
DDR	DNA Damage Repair Response
DLBCL	Diffuse Large B-cell Lymphoma
FAERS	FDA Adverse Event Reporting System
FDA	Food and Drug Administration
FL	Follicular Lymphoma
FNPVD	French National Pharmacovigilance Database
HAECs	Primary Human Aortic Endothelial Cells
HDAC	Histone Deacetylase
hTERT	Human Telomerase Reverse Transcriptase
IRB	Institutional Review Board
LDAC	Low-Dose Cytarabine
MedDRA	Medical Dictionary for Regulatory Activities
mTOR	mammalian Target of Rapamycin
PARP	Poly (ADP-ribose) Polymerase
PRR	Proportional Reporting Ratio
PT	Preferred Term
Q	Quartile
Rb	Retinoblastoma Protein
RCTs	Randomized Controlled Trials
ROR	Reporting Odds Ratio
SAGA	Senescence-associated Growth Arrest
SAHF	Senescence-associated Heterochromatin Foci
SASP	Senescence-associated Secretory Phenotype
SA-β-gal	Senescence-associated β-galactosidase
SLL	Small Lymphocytic Lymphoma
SOC	System Organ Class
TIS	Therapy-Induced Senescence
TNBC	Triple-Negative Breast Cancer
TRF2	Telomeric Repeat-binding Factor 2
WBC	White Blood Cell
